# Is triggering receptor expressed on myeloid cell 1 (TREM-1) protein a new marker of serious infectious complications in colorectal surgery?: case-matched pilot study

**DOI:** 10.1007/s00423-023-03103-z

**Published:** 2023-09-21

**Authors:** Magdalena Pisarska-Adamczyk, Anna Rzepa, Maria Kapusta, Karolina Zawadzka, Beata Kuśnierz-Cabala, Michał Wysocki, Piotr Małczak, Piotr Major, Anna Zub-Pokrowiecka, Mateusz Wierdak, Michał Pędziwiatr

**Affiliations:** 1 Department of Medical Education, Jagiellonian University Medical College, Krakow, Poland; 2 2nd Department of General Surgery, Jagiellonian University Medical College, Jakubowskiego 2, 30-688, Krakow, Poland; 3 Department of Diagnostics, Jagiellonian University Medical College, Chair of Clinical Biochemistry, Krakow, Poland; 4 Doctoral School of Medical and Health Sciences, Jagiellonian University Medical College, Krakow, Poland; 5 Faculty of Medicine, Chair of Medical Biochemistry, Jagiellonian University Medical College, Krakow, Poland; 6 Department of General Surgery and Surgical Oncology, Ludwik Rydygier Memorial Hospital in Krakow, Krakow, Poland

**Keywords:** TREM-1, Colorectal, Surgery, Infectious, Complications

## Abstract

**Purpose:**

The purpose of the study was to evaluate the usefulness of the triggering receptor expressed on myeloid cell 1 (TREM-1) protein as a marker for serious infectious complications during laparoscopic colorectal surgery.

**Methods:**

Sixty-four patients with colon or rectal cancer, who underwent an elective laparoscopic colorectal cancer surgery from November 2018 to February 2020, were included in the analysis. Blood samples of the TREM-1 protein testing were collected four times from each patient: before and on three following postoperative days (PODs). Patients were divided into two groups according to the presence of infectious complications. Subsequently, patients with infectious complications (group 1) were matched 1:1 with patients without complications (group 2). The case-matched analysis was done by selecting patients from the control group by age, ASA scale, cancer stage, and type of surgery.

**Results:**

There was no significant difference in demographic and operative characteristics between the two groups. The median length of hospital stay was longer in group 1 than in group 2 (11 days vs. 5 days, *p* < 0.001). Preoperative measurements of TREM-1 protein did not differ between the two groups. There were no significant differences in the measurements on the first and third postoperative days. However, the median TREM-1 measurement was higher in group 1 on the second postoperative day (542 pg/ml vs. 399 pg/ml; *p* = 0.040). The difference was more apparent when only severe postoperative complications were considered. When compared to the group without any complications, the median TREM-1 level was significantly higher in the group with severe infection complications in POD 1, POD 2, and POD 3 (*p* < 0.05). The receiver operating characteristic (ROC) curve demonstrated that TREM-1 readings in POD 2 had a sensitivity of 83% and a specificity of 84% for the presence of severe infection complications at a value of 579.3 pg/ml (AUC 0.8, 95%CI 0.65–0.96).

**Conclusion:**

TREM-1 measurements might become a helpful predictive marker in the early diagnosis of serious infectious complications in patients following laparoscopic colorectal surgery.

## Introduction

The development of minimally invasive surgical techniques and tailored adjuvant treatment has contributed to a remarkable reduction in morbidity and mortality of patients with colorectal cancer [1]. In particular, the implementation of the Enhanced Recovery After Surgery (ERAS) guidelines has undeniably contributed to improving perioperative outcomes for these patients and reducing the length of hospital stay after surgery [2]. However, the significantly reduced length of stay (LOS), and, therefore, shorter time for close monitoring of the patient, is challenging, as some of the complications develop later than the typical LOS. The symptoms of postoperative infections usually become apparent 4–6 days after surgery [3]. Many studies indicate that infectious complications after colorectal resection still represent a substantial burden. They complicate up to 30% of procedures, contribute greatly to the need for readmission, and have an adverse effect on cancer-specific survival, and their inadequate treatment may promptly lead to sepsis and death [4–6].

Many attempts have been made to find markers that would help to identify patients at particular risk of developing an infection, in order to detect it as soon as possible and implement appropriate treatment. Well-known markers such as C-reactive protein (CRP), interleukin 6, or procalcitonin have been investigated as tools to predict the occurrence of postoperative infectious complications [7, 8]. However, none of them is fully reliable, mainly due to their late rise after surgery [3].

The TREM-1 (triggering receptor expressed on myeloid cell 1) glycoprotein, which belongs to the immunoglobulin superfamily, is a receptor involved in the activation of monocytes and neutrophils during the inflammatory process. There are many reports indicating that the soluble form of this receptor is a reliable diagnostic marker of infection and inflammatory response induced by trauma [9, 10]. TREM-1 has been described as a means to assess the risk of the occurrence of infections in some surgical conditions, but no one has studied its application in predicting complications in patients who underwent elective laparoscopic resection of colorectal cancer [11, 12]. Therefore, we set out to investigate the potential use of soluble TREM-1 (sTREM-1) to predict serious infectious complications in patients undergoing laparoscopic colorectal surgery.

## Materials and methods

Patients with colon or rectal cancer, who underwent elective laparoscopic colorectal cancer surgery between November 2018 and February 2020, were included in the study. Blood samples for the TREM-1 protein assay were collected from each patient four times: preoperatively and on three following postoperative days (PODs). Clinical data and demographic information of patients (age, sex, comorbidities, ASA (American Society of Anaesthesiologists) physical status) were prospectively collected on the database. After the surgery, the database was supplemented with data related to the procedure (type of surgery, operative time, intraoperative blood loss) and treatment results (complications, length of hospital stay (LOS)).

We defined infectious complications as clinical signs of inflammation in different organs with elevated classical inflammatory parameters from blood samples. We included infectious complications involving all organs, resulting directly from surgical site complications, as well as those related to a general complication of hospitalization such as pneumonia. Complications were graded according to the five-grade Clavien-Dindo (CD) classification. We also divided complications into mild (CD 1–2) and severe (CD 3–5).

Patients with infectious complications who formed group 1 were matched 1:1 with patients without complications (group 2). Case-matched analysis was performed by selecting patients for the control group from the group of patients paired by age, ASA scale, stage of cancer, and type of surgery. Since 2012, in our department, the perioperative care of all patients is carried out based on the ERAS protocol, and the laparoscopic approach has been the gold standard in colorectal surgery at our center.

### Inclusion and exclusion criteria

We included adult patients (> 18 years old) with histopathologically confirmed colorectal adenocarcinoma who underwent laparoscopic resection of the colon and/or rectum. Exclusion criteria were as follows: open or emergency surgery, multivisceral resection, stage IV cancer according to American Joint Committee on Cancer (AJCC) classification system, concomitant inflammatory bowel disease, autoimmune systemic disease, other active infection, or when conversion to open resection was necessary.

### Sample size calculation

Due to the lack of previous research using the TREM-1 protein as a marker of infectious complications in surgery and the inability to predict the differences, we assumed that the 10% difference in measured sTREM-1 level represents a clinically relevant difference. Assuming the test power is 90%, we calculated that to detect this, at least 29 patients would be required in each treatment arm.

### Blood samples

Blood samples were drawn four times: on the day of surgery (preoperatively) and on 3 following postoperative days (PODs). Blood was drawn at the same time in the morning, before the meal. Serum from the blood sample (1 vial of 4.9 ml) was centrifuged for 10 min at 4000 rpm and then frozen at −80 °C until all patients were included in the study. Quantikine ELISA Human TREM-1 kit from R&D systems was used. The minimal detectable dose (MDD) of human TREM-1 ranged from 2.65–15.2 pg/ml. The mean MDD was 7.69 pg/ml.

### Statistical analysis

All data were analyzed with Statsoft STATISTICA v.13 (StatSoft Inc., Tulsa, OK, USA). The results were presented as mean ± standard deviation (SD) or median and interquartile range (IQR) when appropriate. Independent sample Student’s *t*-test was used to compare the means of two continuous normally distributed variables, the Mann-Whitney *U* test was used to compare the means of two continuous non-normally distributed variables, and the chi-squared test was used for categorical variables. A receiver operating characteristic (ROC) curve was applied to obtain the area under the curve (AUC) with a 95% confidence interval for AUC and determine the best cut-offs for measurements. Results were considered statistically significant when the *p* value was found to be less than 0.05.

### Ethical approval

All procedures were performed in accordance with the ethical standards laid down in the 1964 Declaration of Helsinki and its later amendments. Informed consent was obtained from all participating patients before surgery. This study was approved by the local ethics review committee (approval number 1072.6120.246.2018) and was registered in the clinicaltrials.gov (NCT05933408).

## Results

During the study period, a total of 136 patients with colorectal cancer were operated in our unit. Eighteen patients did not meet inclusion criteria (benign disease, primary open surgery, stage IV colorectal cancer). Out of the remaining 118 patients, 1 had multivisceral resection; 4 were converted; 2 needed ICU to stay immediately after surgery; 1 patient had concomitant inflammatory bowel disease; in 4 patients, metastases were found intraoperatively; and 2 had autoimmune systemic disease. Out of 104 patients, 32 had infectious complications and were included in group 1. Group 2 consisted of 32 patients without complications.

**Fig. 1:**
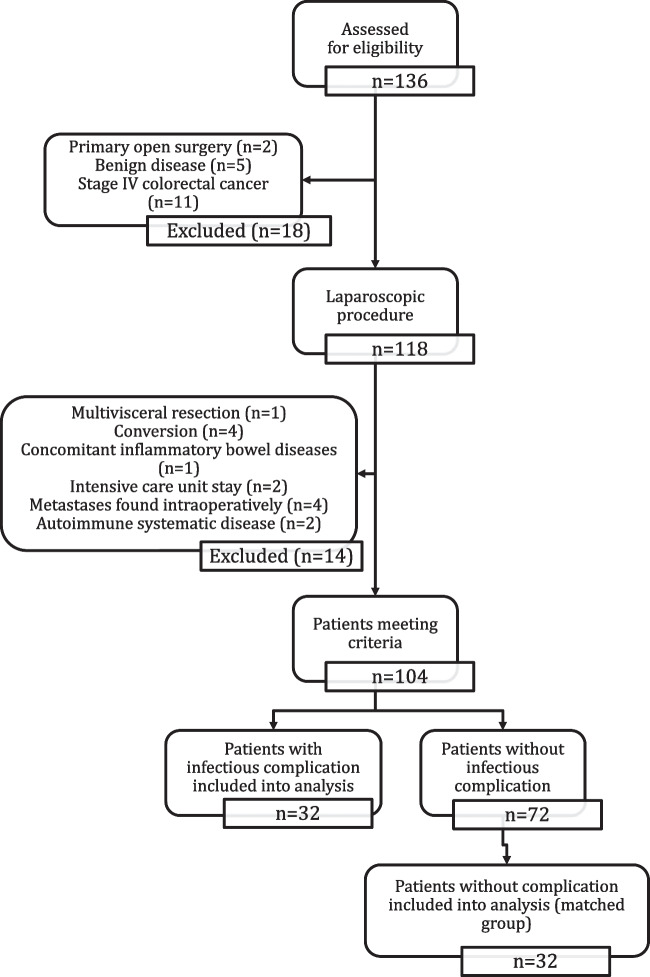
Patients flow through the study

Sixty-four patients were included in the analysis (Fig. 1). Demographic and operative analysis of patient groups is presented in Table 1.

**Table 1:** Demographic and operative analysis of patient groups

Parameter	Group 1 (complicated)	Group 2 (uncomplicated)	*p* value
Number of patients, *n*	32	32	–
Females, *n* (%)	14 (43.8%)	13 (40.6%)	0.801
Males, *n* (%)	18 (56.2%)	19 (59.4%)	
Mean age, years ± SD	67.3 ± 16.2	63.8 ± 11.8	0.266
BMI, kg/m^2^ ± SD	25.9 ± 3.6	26.1 ± 4.5	0.317
ASA 1, *n* (%)	1 (3.1%)	1 (3.1%)	0.855
ASA 2, *n* (%)	21 (65.6%)	23 (71.9%)	
ASA 3, *n* (%)	10 (31.3%)	8 (25%)	
AJCC stage I, *n* (%)	11 (34.4%)	10 (31.3%)	0.875
AJCC stage II, *n* (%)	10 (31.3%)	9 (28.1%)	
AJCC stage III, *n* (%)	11 (34.4%)	13 (40.6%)	
Right hemicolectomy, *n* (%)	11 (34.4%)	12 (37.5%)	0.863
Left hemicolectomy, *n* (%)	3 (9.4%)	2 (6.3%)	
Sigmoid resection, *n* (%)	7 (21.9%)	5 (15.6%)	
TME, *n* (%)	11 (34.4%)	13 (40.6%)	
Mean operative time, min ± SD	229.8 ± 67.7	219.2 ± 70.9	0.5614
Mean intraoperative blood loss, ml ± SD	168.7 ± 131.3	172.9 ± 155.3	0.9444
Median length of hospital stay (days, IQR)	11 (7–17)	5 (4–6)	< 0.0001

Of the group of 32 patients with complications, serious complications occurred in 13 of them (Table 2). The most common serious complication was anastomotic leak requiring surgery, which occurred in 7 patients. In 2 patients, the anastomotic leak was successfully treated endoscopically with endo-VAC. Other Clavien-Dindo grade 3 complications include pelvic abscess and peristomal fistula. One patient required an ICU stay due to pneumonia that occurred after reoperation caused by intestinal perforation. One patient died in the ICU due to pneumonia leading to respiratory failure.

**Table 2:** Types of complications

Clavien-Dindo classification		Complications	
Severe complication (CD 3–5)	5	Pneumonia leading to respiratory failure, death in the ICU	1/32
	4	Peritonitis and reoperation due to intestinal perforation, pneumonia, ICU stay	1/32
	3 B	Peristomal fistula	1/32
		Anastomosis leakage (relaparoscopy, laparotomy)	7/32
	3 A	Pelvic abscess	1/32
		Anastomosis leakage (treated with endo-VAC)	2/32
Light complication (CD 1 and 2)	2	Surgical site infection (vacuum-assisted therapy)	2/32
		Fever with prolonged ileus requiring TPN	1/32
		Pneumonia	3/32
		Urinary tract infection	2/32
	1	Surgical site infection	2/32
		Fever of unknown origin	4/32
		Infectious diarrhea	2/32
		Delayed healing of anastomosis	3/32

Preoperative measurements of TREM-1 protein did not differ between groups. There were no significant differences in measurements between those groups on the first and third postoperative days. Median TREM-1 measurement was significantly higher in group 1 on the second postoperative day (542 vs. 399 pg/ml; *p* < 0.05), respectively. The difference was more noticeable when only severe postoperative complications were taken into consideration. Median TREM-1 level was significantly higher in the group with severe infectious complications when compared to the group without any complications in PODs 1 (*p* < 0.05), 2 (*p* < 0.05), and 3 (*p* < 0.05). Details of the biochemical analysis are provided in Table 3.

**Table 3:** Analysis of biochemical parameters

Parameter		Group 1 (patients with infectious complications)	Group 2 (patients without complications)	*p* value
Median TREM-1 (IQR) (pg/ml)	POD 0	296 (215.8–360.4)	306.2 (241.1–392.6)	0.432
	POD 1	498.9 (394.5–559.3)	442.2 (347.8–524.7)	0.134
	POD 2	542.2 (386.8–666.37)	398.8 (304.9–515.8)	< 0.05
	POD 3	396.8 (280.8–656.1)	306.9 (238.5–386.5)	0.074
		Patients with severe complications (CD 3–5)	Group 2 (patients without complications)	*p* value
Median TREM-1 (IQR) (pg/ml)	POD 0	357.3 (334.6–379.1)	306.2 (241.1–392.6)	0.408
	POD 1	569.8 (521.4–816.9)	442.2 (347.8–524.7)	< 0.05
	POD 2	710.6 (583.8–805.5)	398.8 (304.9–515.8)	< 0.05
	POD 3	688.5 (410.1–853.5)	306.9 (238.5–386.5)	< 0.05

Also, the increase in sTREM values from baseline on consecutive days was significant in subsequent PODs. The differences were statistically significant on all days between the group of uncomplicated patients and those with severe infectious complications (Table 4).

**Table 4:** Analysis of biochemical parameters

Parameter		Group 1 (patients with infectious complications)	Group 2 (patients without complications)	*p* value
Median TREM-1 (IQR) (pg/ml)	POD 1-0	170 (104.5–229.2)	115.3 (53.7–218.7)	0.214
	POD 2-0	163.7 (91.2–300.8)	80 (15.7–163.3)	< 0.05
	POD 3-0	104.4 (40.9–208.7)	51.9 (−19.7–101.7)	0.084
		Patients with severe complications (CD 3–5)	Group 2 (patients without complications)	*p* value
Median TREM-1 (IQR) (pg/ml)	POD 1-0	201.2 (97.9–441.3)	115.3 (53.7–218.7)	< 0.05
	POD 2-0	330.7 (221.8–405.2)	80 (15.7–163.3)	< 0.05
	POD 3-0	312 (69.20–293.4)	51.9 (−19.7–101.7)	< 0.05

**Fig. 2:**
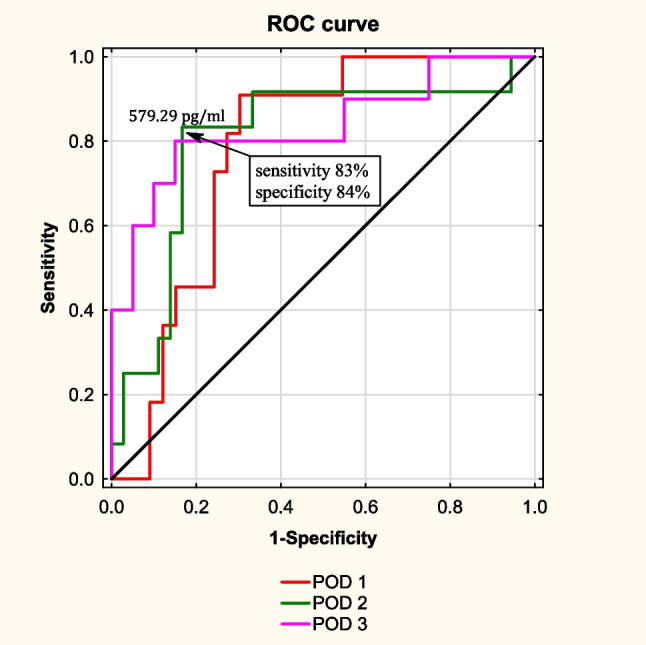
Receiver operating characteristic (ROC) curve to determine the optimal cut-off of TREM-1 measurements for the occurrence of severe infectious complications

The analysis showed that measurements on POD 2 were characterized by the most favorable ratio of sensitivity to specificity. The receiver operating characteristic (ROC) curve showed that TREM-1 measurements in POD 2 had a sensitivity of 83% and a specificity of 84% for value 579.3 pg/ml (AUC 0.8, 95%CI 0.65–0.96) to predict severe infectious complications. Figure 2 shows the ROC curves.

## Discussion

In our study, serum TREM-1 levels were significantly higher in POD 2 in patients who developed infectious complications after surgery than in uncomplicated cases. Furthermore, in patients with severe complications (3–5 on the Clavien-Dindo scale), the rise in sTREM-1 levels was even more pronounced and occurred in PODs 1–3. Importantly, the analysis showed that measurements on POD 2 were characterized by the most favorable ratio of sensitivity to specificity (sensitivity of 83% and specificity of 84% for value 579.3 pg/ml).

Postoperative infectious complications are still a major burden in colorectal surgery as they occur in up to one-third of patients after elective operation and are associated with decreased survival [13]. Their accurate and early prediction could limit morbidity, and possibly improve patients’ long-term outcomes. Indeed, it is particularly important in the present era of minimally invasive surgery and ERAS protocol, when the time spent in the hospital after surgery has been significantly shortened and patients are discharged before most infection complications occur [3].

Due to the shortening of the period of stay and reduction of surgical trauma, many authors have been looking for plasma inflammatory markers as preclinical rapid predictors of infectious complications. To date, the most commonly used marker for screening the development of postoperative infections is C-reactive protein (CRP). Despite the established position of CRP as a protein associated with inflammation, findings regarding its accuracy in predicting complications in colorectal surgery vary widely between studies. For example, some authors have shown that in POD 4, CRP has only 44.4% sensitivity and as much as 98.5% specificity, while others reported 94.4% sensitivity and 64.6% specificity on that day [7, 8]. In addition, CRP is a non-specific marker of inflammation, its levels always rise after the operation as a result of a surgical stress response, and only sustained increased levels of POD 3 or POD 4 have predictive value in predicting infectious complications [14]. Some authors have therefore compared the diagnostic accuracy of PCT with that of CRP in the context of elective colorectal surgery. The conclusion was that the PCT determination is associated with much higher costs, and PCT is no better than CRP in this setting due to its low specificity [7, 14]. Another cytokine produced in response to various inflammatory mediators is interleukin 6 (IL-6). While Wierdak et al. reported a remarkably high ability to predict infectious complications, especially on POD 2 (91% sensitivity and 97% specificity), other studies have not supported this finding [3, 15, 16]. The main drawbacks of the IL-6 assay are its short half-life, which results in high variability in its concentration in patients’ blood, and the profound impact of chronic inflammatory diseases on its concentration [15]. As none of the well-known inflammatory proteins, such as CRP, PCT, or IL-6, are fully sufficient to predict the early occurrence of infectious complications after colorectal surgery, research into new markers that will have better diagnostic value is still needed.

The discovery of additional pathways used by host cells to recognize bacterial components appears promising. The glycoprotein TREM-1 is a receptor involved in the activation of monocytes and neutrophils in the inflammatory process. The literature indicates that soluble TREM-1 is an effective indicator to evaluate the severity and prognosis of infectious diseases [17, 18]. In addition, the concentration of sTREM-1 predicted septic complications better than CRP or PCT [19, 20]. Gonzalez-Roldán et al. showed that there was a significant difference in sTREM-1 blood levels between healthy volunteers and patients after elective gastrointestinal surgery, as well as between the uncomplicated surgery group and patients who developed sepsis [11]. Two studies in patients undergoing cardiac surgery confirmed that high sTREM-1 plasma concentrations correlated with the risk of infectious complications [12, 21]. Interestingly, patients who later developed complications showed a remarkable rise in sTREM-1 already at the end of surgery. Similarly to our study, a significant difference between the group with infectious complications and the group without complications occurred on POD 2 [12].

To the best of our knowledge, we were the first to show that monitoring sTREM-1 is a valuable tool in the early prediction of infectious complications of elective colorectal surgery. We are aware of the limitations of this study; however, it was designed to assess the usefulness of TREM-1 as a predictor of infectious complications in elective minimally invasive colorectal cancer surgery. The study sample is relatively small, and to reliably establish cut-off values, sTREM-1 should be tested in a larger cohort of patients undergoing laparoscopic colorectal surgery. Another limitation is that we did not analyze whether sTREM-1 levels also increase in non-infectious complications, but it has been suggested that there is no significant difference in TREM-1 levels between postoperative patients who developed an infection and surgical patients who developed a non-infectious complication [22].

## Conclusion

The results of the present study indicate that sTREM-1 has the potential to be a valuable predictive marker in the early detection of severe infectious complications in patients undergoing laparoscopic colorectal surgery. In contrast to other widely used inflammatory markers, the level of sTREM-1 rises notably already on the first postoperative day in the group who have experienced serious complications. Our findings are of importance as monitoring the sTREM-1 level could identify patients who have a high risk of developing infectious complications after surgery and should stay in the hospital longer than a typical LOS lasts.
